# Respiratory Changes in Response to Cognitive Load: A Systematic Review

**DOI:** 10.1155/2016/8146809

**Published:** 2016-06-14

**Authors:** Mariel Grassmann, Elke Vlemincx, Andreas von Leupoldt, Justin M. Mittelstädt, Omer Van den Bergh

**Affiliations:** ^1^Department of Aviation and Space Psychology, German Aerospace Center (DLR), Sportallee 54a, 22335 Hamburg, Germany; ^2^Research Group on Health Psychology, University of Leuven, Tiensestraat 102, 3000 Leuven, Belgium

## Abstract

When people focus attention or carry out a demanding task, their breathing changes. But which parameters of respiration vary exactly and can respiration reliably be used as an index of cognitive load? These questions are addressed in the present systematic review of empirical studies investigating respiratory behavior in response to cognitive load. Most reviewed studies were restricted to time and volume parameters while less established, yet meaningful parameters such as respiratory variability have rarely been investigated. The available results show that respiratory behavior generally reflects cognitive processing and that distinct parameters differ in sensitivity: While mentally demanding episodes are clearly marked by faster breathing and higher minute ventilation, respiratory amplitude appears to remain rather stable. The present findings further indicate that total variability in respiratory rate is not systematically affected by cognitive load whereas the correlated fraction decreases. In addition, we found that cognitive load may lead to overbreathing as indicated by decreased end-tidal CO_2_ but is also accompanied by elevated oxygen consumption and CO_2_ release. However, additional research is needed to validate the findings on respiratory variability and gas exchange measures. We conclude by outlining recommendations for future research to increase the current understanding of respiration under cognitive load.

## 1. Introduction

Everyday life experience shows that playing a video game, learning how to drive a car, doing math homework, or performing another cognitively demanding task may affect breathing. In some cases respiration tends to be inhibited; in other cases it seems to accelerate and/or its volume changes. In general, little is known about respiratory alterations under cognitive load. As a consequence, it is not clear whether respiration could be used as a reliable indicator of cognitive load. In human factors and ergonomics, the investigation of cognitive load or “mental load” aims at predicting operator performance (e.g., pilot selection) and optimizing working conditions (e.g., cockpit design) in order to enhance performance and comfort. For this purpose, physiological parameters have been considered valid indicators of cognitive load as they are hypothesized to reveal task-related arousal states in the operator.

Mental load is assumed to be high when the required resources for a satisfactory task completion exceed operator capacity [1–3]. Importantly, the concept of mental load should be differentiated from mental stress. As pointed out by Gaillard and Wientjes [4], arousal due to mental stress is characterized by negative feelings such as anxiety or frustration while mental load is accompanied by neutral or even positive feelings as being challenged. Both concepts assume that the individual experiences some discrepancy between environmental demands and one's coping resources and initiates extra effort. Under mental load, the individual focuses on accomplishing the task and on performance monitoring whereas under stress, the individual is mainly concerned with threats and protection of the self. In the literature, however, the two concepts are often confounded in terms of terminology or experimental implementation [5].

For the manipulation of mental load, researchers typically apply cognitive tasks such as mental arithmetic or Stroop tests. These tasks have in common that they involve several aspects of cognitive processing such as perception, controlled attention, reasoning, memory, problem solving, decision making, and inhibitory control, as well as cognitive control of speech and motor activity inasmuch as this is required for performance. Study designs differ in whether a concurrent performance feedback is provided or not. On the one hand, researchers argue that permanent feedback is necessary for an individual's monitoring of the process and a corresponding regulation of energetic state [6, 7]. On the other hand, performance feedback while accomplishing the task may cause psychological stress in addition to the cognitive task demands [4, 8, 9].

Investigating the concept of mental load dates back to the 1970s [10] and has generated a broad number of different methods that are generally categorized into self-report measures, performance-based measures, and physiological measures [3]. In the past, self-report measures have often been regarded as less reliable and valid than “objective” performance scores and physiological data [1], but today's prevailing view considers the different methods as reflecting different aspects of operator load. As a consequence, present research and real-life assessments in human factors and ergonomics are usually based on a combination of self-report, performance, and physiological measures.

Existing reviews on physiological correlates of cognitive load show that research efforts have mainly been devoted to cardiovascular, electrodermal, and brain activity measures [11–14], while comparatively less research has investigated whether and how respiration is sensitive to cognitive load. Respiration is the biggest oscillator in the body that is involved in regulating processes in response to environmental demands and in maintaining homeostasis. The respiratory rhythm is known to be generated by pacemaker neurons which are located in the lower brainstem [15, 16]. Respiratory activation not only indexes metabolic changes but also psychological and behavioral processes [7]. For example, during cognitive as well as emotional demands, the respiratory rhythm is impacted by suprapontine influences, reflecting also limbic and paralimbic influences [17–19]. While many of the available studies on respiration applied rudimentary measurement techniques [7], more recent studies have adopted assessment methods from respiratory physiology and integrated more sophisticated parameters providing additional insights into breathing behavior under cognitive load (e.g., variability measures, see [20]).

In general, research in respiratory psychophysiology in healthy populations is based on measures reflecting time, volume, and gas exchange aspects of breathing. The most common parameters are respiratory rate (RR) and respiratory amplitude which corresponds to tidal volume (TV), the amount of air that is inspired during one respiratory cycle. Minute ventilation (MV) refers to the amount of air that is inhaled in one minute and is hence contingent upon RR and TV. Further time and volume parameters that are analyzed frequently are inspiratory time (*T*_*i*_) and expiratory time (*T*_*e*_), as well as inspiratory volume (*V*_*i*_), which equals TV and expiratory volume (*V*_*e*_), and the timing ratio of inspiration to expiration (*T*_*i*_/*T*_*e*_). Also mean inspiratory flow rate (TV/*T*_*i*_) and inspiratory duty cycle (*T*_*i*_/*T*_tot_) are occasionally calculated, both quotients indicating the activity of underlying respiratory drive mechanisms (see [21]). Specific response measures such as sigh rate (SR) and the proportion of ribcage breathing to *V*_*i*_ (% RCi) have rarely been reported in the literature. In addition to basic time and volume parameters, corresponding variability measures have been computed to quantify total variability by using statistical variance (Var) or the coefficient of variation (CV) as well as structured variability by using the autocorrelation (AR) of successive breaths. Since total variability is considered to comprise structured and random portions [20, 22], which might be affected differently by environmental demands, total variability measures should be interpreted together with a measure of correlated variation. Among the gas exchange parameters, partial pressure of end-tidal carbon dioxide (petCO_2_), an estimate of arterial pCO_2_, is particularly interesting since reduced CO_2_ values generally indicate that ventilation is in excess of metabolic need. Also oxygen consumption (VO_2_) and CO_2_ production (VCO_2_), which usually covary with MV, as well as the proportion of released CO_2_ to inhaled O_2_ (respiratory exchange ratio, RER) have been investigated to determine energy expenditure in demanding situations.

Since most of the outlined measures may vary with age, gender, and physical fitness, it is useful to take possible control variables into account when investigating respiratory reactivity in healthy individuals [23–26]. Apart from person-related covariates, verbal activity during data acquisition can influence time and volume parameters. Speech production requires a coordination of articulatory and respiratory movements which can override the regular respiratory rhythm [27, 28] and typically leads to a shorter *T*_*i*_, accompanied by increased airflow velocity, and to a longer expiratory time together with decreased airflow velocity [29, 30]. In addition, speech and motor activity can cause artifacts in the recording process. While spirometric and capnographic methods directly sampling from mouth and nose are inevitably affected by vocal activity, electronic signals of impedance-based methods are particularly prone to motion [31, 32]. As a consequence, most researchers counter such artifacts by selecting tasks that require a minimum of speech and motor activity and by instructing participants not to talk or move during the periods of data acquisition, unless it is required.

General measurement techniques to quantify respiration in healthy individuals comprise spirometry, respiratory inductive plethysmography, strain gauges, impedance-based methods, capnography, and metabolic analyzers. While all these techniques are usually suited to record timing parameters, the amplitude of breathing can only be assessed by a direct measurement of lung volume (using spirometry) or indirectly through changes in girth of thorax and abdomen (using strain gauges or respiratory inductive plethysmography) or through changes in impedance of the thorax. Spirometric devices such as spirometers, flowmeters, and pneumotachographs provide accurate assessments of *V*_*i*_ and *V*_*e*_ but also require participants to wear a facemask, a mouth- or noseclip that, in itself, may alter the respiratory behavior [33]. Most common is the use of inexpensive strain gauges, converting mechanical strain into voltage, and inductive plethysmography, measuring self-inductance in transducer bands. Both techniques are unobtrusive and easy to handle. However, without a constant and valid calibration procedure they do not provide absolute measures of respiratory depth. The same applies to impedance plethysmography which additionally is rather expensive. Hence, it has been suggested to estimate RR and amplitude by means of spectral analysis from the impedance cardiography signal [34–36] since respiratory and cardiac monitoring are often combined. PetCO_2_ is mainly assessed by means of a capnograph with infrared spectrography and a sampling site that is attached to the mouth and/or nose. For the combined determination of VO_2_ and VCO_2_, metabolic measurement systems are used which commonly are equipped with paramagnetic O_2_ and infrared CO_2_ sensors. Due to the direct sampling from mouth and nose, also capnographs and metabolic analyzers are rather intrusive but, on the other hand, they provide accurate and absolute assessments of respiratory parameters.

The objective of this review was to provide an overview on empirical studies examining the respiratory effects of cognitive load for research and application purposes such as monitoring operator load. Specifically, we aimed at analyzing all published results provided by a search in electronic databases that investigated changes in at least one respiratory measure from a baseline to a task period characterized by any kind of cognitive load. We further integrated findings on respiratory sensitivity to different levels of task difficulty as well as findings on the effect of task duration. Finally, we made a comparative evaluation of respiratory reactivity under experimental conditions with and without concurrent performance feedback.

## 2. Literature Research and Study Selection

Electronic database searches of PsycINFO, PubMed, and Web of Science were conducted using the following terms without a priori publication date restrictions:

 [cognitive OR mental OR attentional] AND [load OR workload OR stress OR effort] AND [respirat^*∗*^ OR breath^*∗*^ OR CO_2_].

This query yielded 819 references. After an examination of title and abstract, 636 irrelevant sources were excluded. The remaining 183 references were subjected to a detailed screening based on the full papers. We selected journal publications in English language reporting original data on respiratory measures in response to cognitive task load. Study samples had to consist of healthy adult participants breathing spontaneously during at least one period of data acquisition under cognitive load. Clinical studies and experiments that entailed physical activity, emotion induction, physical or psychosocial stress (e.g., cold pressor, public speech), manipulated or controlled breathing, or pharmaceutical intervention were only selected if respiratory data were reported for a control group or control condition (i.e., spontaneous breathing without any manipulation other than cognitive load), respectively. For those studies, only data from the control group and/or control condition were taken into account. Further inclusion criteria were data acquisition during rest for the analysis of baseline-to-task changes as well as a limited level of movement activity and speech, which might in itself impose respiratory changes. Strictly speaking, studies were excluded if participants were allowed to move and/or to speak more than briefly indicating a required task response by moving a mouse cursor or saying a single word or number.

The study selection was conducted independently by two authors to ensure reliable data acquisition [37]. If authors disagreed, the procedure was repeated for the corresponding reference after discussing the prevailing concern. References with divergent ratings after the second screening were classified through consensus discussion.

## 3. Data Extraction and Synthesis

A total of 53 journal articles evaluating respiratory parameters in response to cognitive task load and meeting the selection criteria were included in this review. Study characteristics and findings were extracted from every publication and listed. During the process of data acquisition and integration, this list was completed with additional variables that appeared relevant and eventually covered the following information: first author, publication year, sample size and characteristics, type of experimental manipulation, duration of analyzed task period, respiratory outcome measures and measurement techniques, additional information on verbal activity and performance feedback, and the reported findings on respiratory changes in response to cognitive task load including possible effects of task difficulty and duration (if analyzed).

### 3.1. Study Characteristics

Table 1 summarizes the study characteristics of 54 experiments which are reported in 53 articles. Sample sizes ranged between 7 and 132 participants with a mean of 32 participants. The experimental tasks to induce cognitive load were categorized according to the cognitive processes primarily required for accomplishing the task (i.e., attention, reasoning, short-term or working memory, psychomotor coordination, and vigilance) or according to the respective task type/paradigm if more than one major facet of cognitive control was required (i.e., Stroop, mental arithmetic, choice reaction time, and multitasking). Operator load was most often manipulated by administering mental arithmetic or multiple tasks, followed by attention, memory, Stroop, reasoning, and psychomotor tasks. As depicted, RR is the only variable that has been analyzed in all studies under review. Apart from TV and MV, all other parameters have been evaluated in fewer than seven studies. To collect respiratory signals, most of the studies used a strain gauge or an inductive plethysmograph, which are not intrusive and relatively easy to handle. However, spirometry, capnography, and impedance plethysmography were also conducted in at least five of the reviewed studies.

A detailed list of the studies included in the present review is shown in Table 2. As indicated, the duration of task period that was extracted for data analysis varied between 30 and 1800 sec, with 57% of the studies choosing sampling periods lasting between 180 and 300 sec. Four of the seven studies requiring a verbal task response systematically investigated the effect of verbal activity on respiratory changes under cognitive load [38–41]. A concurrent performance feedback was given in 35% of the studies. One of these studies systematically compared respiratory reactivity to cognitive task load with and without performance feedback [7].

### 3.2. Coding and Integration of Effects

To integrate the findings on respiration under cognitive load, we coded baseline-to-task changes for every respiratory outcome measure as increasing (↑), decreasing (↓), or not significantly changing (—) at *p* < .05. If a study included more than one experimental period (trial), reported findings were counted according to the number of trials (e.g., twice if a study reported an increase in two trials).  Table 3 displays overall effects as reported by more than 50% of the reviewed studies. If two different effects were revealed by an equal number of studies or experimental periods (e.g., increase and decrease were found in five studies each), we coded the overall effect accordingly as ↑↓, ↑—, or ↓—. The same procedure was applied to review respiratory changes in response to different levels of task difficulty (*n* = 14) as well as changes in respiratory reactivity over time (*n* = 6).

**Figure 1:**
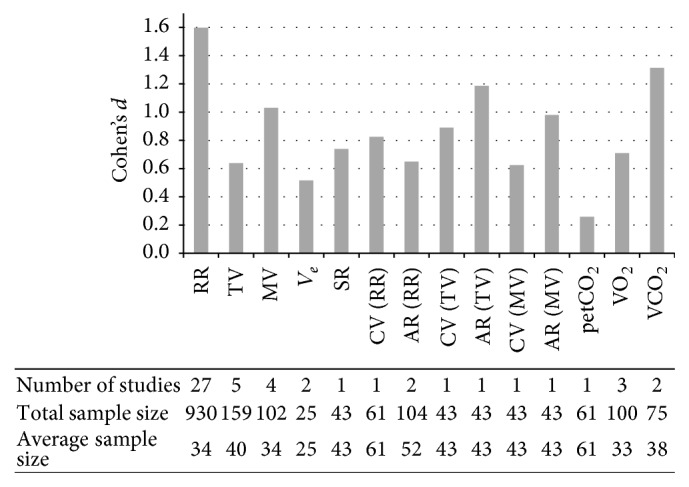
Sample weighted means of Cohen's *d* for each respiratory parameter considering all studies that provided the required descriptives to compute standardized mean differences. The respective number of studies as well as total sample size and average sample size are displayed in the column below each parameter (RR: respiratory rate; TV: tidal volume; MV: minute ventilation; *V*_*e*_: expiratory volume; SR: sigh rate; CV: coefficient of variation; AR: autocorrelation; petCO_2_: partial pressure of end-tidal carbon dioxide; VO_2_: oxygen consumption; VCO_2_: carbon dioxide production).

In order to evaluate the magnitude of effects, we computed standardized mean differences for every significant baseline-to-task change for which the required descriptive data were available. In sum, 50% of the reviewed studies reported their significant findings together with mean (M) and standard deviation (SD) or standard error (SE) scores for either baseline and task conditions or for the discrepancy between baseline and task. As a measure of effect size, we calculated whenever feasible Cohen's *d* for each respiratory parameter using pooled SD and adjusted for small sample bias if less than 50 participants were included in the reported analysis [89]. For a comparative summary of the obtained effect sizes, we additionally computed a sample weighted mean effect size for each measure [90, 91]. As illustrated in Figure 1, the most robust measure is RR which is based on a total sample of 930 individuals. A solid data base yielded by more than three studies was further available for TV and MV, showing that strong effects are also reported for MV while TV should not be regarded as a robust measure. In the following, we present the integrated effects for each respiratory measure. Corresponding effect sizes are reported and evaluated if (a) the overall effect for the respective measure indicates an increase or decrease and (b) at least one study confirming to the overall effect was available for calculating Cohen's *d*.

### 3.3. Empirical Findings on Respiration and Cognitive Load

As shown in Table 2, the breathing pattern under cognitive load was mainly characterized by faster respiration than during baseline. Following the guideline by Cohen [92], five studies suggest small effects (.20 ≤ *d* < .50 [20, 38, 45, 47, 88]), six studies suggest medium effects (.50 ≤ *d* < .80 [20, 47, 60, 61, 66, 69]), and 20 studies suggest large effects (*d* ≥ .80 [38, 41, 42, 45, 46, 51, 54, 58, 63–66, 68, 73, 76, 79, 80, 82, 87] (two studies reported in [73])) for the increase in RR. For one study, the calculated effect size indicates that the significant increase in RR while performing a vigilance task can be considered negligible (*d* = .12 [76]). Table 2 further shows that higher task difficulty resulted in an additional increase of RR. Those studies providing data for calculating effect sizes on different levels of task difficulty indicate that the increase in RR changed from small to medium effects [47] and from medium to large effects [45, 66] when the task became more difficult in a parallel fashion.

While the studies including TV are rather inconsistent and mainly reported no significant changes from baseline to task, MV increased in all studies with two studies suggesting large effects [42, 66] and one study suggesting a small effect [20] for the increase in MV. MV was not related to varying difficulty levels. Overall, reactivity patterns were further marked by a reduced correlated variability (AR) of RR with two studies indicating medium effects [20, 58], whereas total variability (Var, CV) of RR was mostly invariant to cognitive load. Capnographic measures show that petCO_2_ levels were lower during task performance (*d* = −.26; [58]), while VO_2_ and VCO_2_ were higher during the task and VCO_2_ additionally elevated with increasing difficulty. Effect sizes for the increase in VO_2_ ranged from small [46, 63] to medium and large effects [42] and from medium [47] to large effects [42] for the increase in VCO_2_. The available data suggest that both low and high levels of task difficulty elicited a medium increase in VCO_2_ from baseline to task [47]. The two studies including RER revealed opposite results, one increasing and one decreasing from baseline to task. Habituation effects were only reported for RR. However, the same number of studies provides support that elevations in rate persisted over time or from trial to trial. Across all trials, the analyzed task period averaged 1550 sec for the studies indicating habituation and 2270 sec for the studies reporting no change in reactivity.

Respiratory reactivity effects published in the seven studies that required verbal responding to the cognitive task [38–40, 47, 50, 70, 88] largely correspond with the overall effects: an elevated RR (available effect sizes are provided below) and no change in respiratory waveform (*T*_*i*_/*T*_*e*_). TV, however, was found to decline from baseline to tasks with verbal responding in one out of three studies analyzing TV [88]. The only study including respiratory variability reported a decrease in total variability of RR, which is not in line with the overall effects outlined above [70]. In addition, it has to be noted that the systematic investigation revealed significant differences between “silent” and “aloud” conditions in three out of four studies: In one study, RR was found to be increased only in the “aloud” condition of a Stroop interference task (*d* = 6.40 [38]) while in two other studies, rate was found to be increased only in the “silent” condition of a mental arithmetic task (*d* n/a [39], *d* = 1.88 [41]). Furthermore, a reduced TV has been reported exclusively when participants remained silent [39], which conflicts with the finding mentioned above [88], as well as a reduced *T*_*i*_/*T*_*e*_ when participants indicated their response verbally (*d* n/a [39]).

When comparing the respiratory measures from experimental conditions with and without concurrent performance feedback that were investigated by a minimum of three studies, we found for both conditions an increase in RR and in MV from baseline to task. Effect sizes were available for ten studies with at least one feedback condition, suggesting small [20, 47, 88] as well as medium [20, 47, 60, 61, 69] and large effects [51, 68, 80, 87] for RR and small [20] as well as large [41] effects for MV. The available 16 studies with at least one no-feedback condition mainly suggest large effects for RR [38, 41, 42, 45, 46, 54, 58, 63–66, 73, 76, 79, 80] and MV [42]. Interestingly, the implementation of feedback was mostly associated with a decrease in TV, two studies indicating small effects (*d* = −.27 [7], *d* = −.26 [20]) and one indicating a large effect (*d* = −2.51 [50]), whereas the experiments not providing feedback did not show significant changes in TV. The direct comparison of feedback and no-feedback conditions within a single experiment revealed no significant differences in reactivity for any respiratory parameter under study (RR, TV, MV, TV/*T*_*i*_, *T*_*i*_/*T*_tot_, and petCO_2_ [7]).

## 4. Discussion

This study was conducted to review the available literature on respiration under cognitive load by integrating findings on respiratory changes from baseline to task and possible effects of task difficulty, task duration, and concurrent performance feedback. In addition, we surveyed the methods used to manipulate cognitive load and to quantify respiration and separately analyzed respiratory changes from baseline to tasks that required verbal responding.

### 4.1. Respiratory Responses to Cognitive Load

The present findings show that cognitive load was accompanied by a clear increase in RR. Of note, 48% of the reviewed studies indicated medium to large effects for the increase from baseline to task. Also, higher levels of task difficulty resulted in an additional increase of RR. While TV appeared to be insensitive to cognitive load, MV, following logically from the increase in RR without changes in TV, showed a consistent increase from baseline to task. Since MV was, however, not sensitive to different levels of task difficulty and predominantly reflects the increase RR, we conclude that, for the assessment of operator load, MV does not provide incremental information over the more convenient frequency measure. This general increase in ventilation has been explained by a higher metabolic rate during performance [47] but also by psychological processes such as learned anticipation of metabolic need [93–95]. While human and animal research on limbic and paralimbic influences on breathing is still scarce, available evidence suggests that an increase in cognitive as well as emotional impact is associated with corresponding changes in neural activity not only in the brainstem but also in the limbic and paralimbic regions, particularly amygdala and anterior cingulate cortex [17, 96], the latter being a key prefrontal region that is also involved in executive function [97–99].

The timing parameters discussed in the following have been investigated by less than three studies and should thus be interpreted with caution. Considering the increase in RR, a shorter *T*_*i*_ and an invariant ratio of *T*_*i*_ to *T*_*e*_ signify a shortening of both *T*_*i*_ and *T*_*e*_ under cognitive load. The reported increase in TV/*T*_*i*_ was observed together with no changes in *T*_*i*_/*T*_tot_ [7], indicating that the overall elevations of ventilation are rather caused by a higher “intensity of the central inspiratory drive mechanism” ([21, p. 106]) than by alterations in timing. However, Pattyn et al. [73] provide support that also the timing mechanism might trigger the increase in ventilation under cognitive load, suggesting that additional studies are needed to clarify the underlying mechanisms of ventilatory changes.

Frequency of sighing under cognitive load was investigated by two studies [20, 86] showing that SR increased in response to mental arithmetic. The authors assume that sighing counteracts erratic breathing patterns which may occur under cognitive load. Also in the present study, cognitive load consistently elicited a decrease in correlated variability of RR while, overall, total variability of RR did not change from baseline to task. This implies that random variability tends to increase when performing a cognitive task. Evaluating variability measures of TV and MV mainly revealed no changes from baseline to task. Only total variability of TV has been reported to increase considerably during mental arithmetic [20, 86].

Moreover, cognitive load was shown to be associated with reduced petCO_2_, indicating hyperventilation, and higher levels of VO_2_ as well as VCO_2_, which are usually assessed to track energy expenditure. The decrease in petCO_2_ and the increase in VCO_2_ appear to be conflicting. It has to be noted, however, that petCO_2_ is a fractional measure, not allowing conclusions about absolute CO_2_ levels, and that the relationship between etCO_2_ and VCO_2_ is contingent on alveolar ventilation which could have differed between the respective samples. VCO_2_ is the only capnographic measure that demonstrated medium to large effect sizes and was sensitive to increasing task difficulty, which suggests that mental effort actually entails additional energy expenditure (see also [47]). PetCO_2_ was less sensitive regarding the magnitude of changes and task difficulty. However, petCO_2_ was consistently reported to decrease whereas findings on VCO_2_ did not entirely point in the same direction. Since petCO_2_ additionally provides information on whether ventilatory changes in response to cognitive load correspond to actual changes in metabolic demand, both petCO_2_ and VCO_2_ are promising indicators of cognitive load. Future studies should therefore validate and integrate the existing findings.

Only six studies analyzed respiratory changes over the course of the cognitive task or from trial to trial. For most variables, these studies revealed no change as well as inconsistent findings for the habituation of RR. Hence, changes in respiratory reactivity over time also require further investigation.

### 4.2. Methodological Evaluation

By specifying a priori selection criteria, we obtained a database of experimental studies on cognitive load with an acceptable degree of consistency regarding study design and the induction of operator load. Although performance feedback during cognitive tasks has been assumed to elicit stress responses comparable to the effects of social evaluative threat [4, 100, 101], we decided not to exclude these studies but to analyze them additionally in comparison with studies not providing feedback. Also, we did not exclude any study applying verbal-response tasks in order to maintain a sufficiently large sample of studies. Instead, we accepted studies with a limited amount of verbal activity and additionally evaluated them separately in a subanalysis.

As summarized above, most of the studies manipulated cognitive load by means of mental arithmetic or multitasking. The 11 studies including more than one type of cognitive task in their experimental design (see Table 2) imply that the magnitude of respiratory effects may vary depending on the given types of cognitive demands. Particularly sigh rate and respiratory variability measures have been shown to be differently altered by mental arithmetic and attentional tasks [20, 70, 86]. However, the current database is insufficient for a systematic investigation of respiratory responses to various facets of cognition. Also the findings reported by Roman-Liu et al. [76] suggest that vigilance behavior, involving uninterrupted attention on the detection of infrequent signals, is characterized by particular respiratory changes which should be addressed in future studies.

In a separate analysis, we investigated whether concurrent performance feedback affects respiratory responses to cognitive load. In general, the reviewed studies imply that performance feedback on cognitive tasks is only accompanied by a decrease in TV, which has not been observed in the overall findings outlined above. Since decreases in TV have also been reported for negative emotions such as anxiety and sadness [102], we conclude that a concurrent feedback may have emotional impact on the operator, inducing stress rather than mental load (see also [4, 8, 9]), and should thus be avoided in future experiments on cognitive load. Unfortunately, no data were available to evaluate the effect of performance feedback on variability measures.

Our survey revealed that most of the studies used recording techniques that are not directly disturbed by speech. But since respiration itself may be affected by verbal activity, we additionally reviewed the seven studies involving speech. In sum, the findings were inconsistent and generally corresponded to the overall effects. However, the systematic comparison of verbal- and manual-response conditions also revealed some indication for erratic breathing patterns as well as shorter inspiration and longer expiration phases when performing a mental arithmetic with verbal responding. Given that this is in line with the existing knowledge about the interplay of speech and respiration [29, 30] and that manual responding is easy to integrate in computerized task designs, we suggest evaluating respiratory measures for the assessment of operator load only under silent conditions.

### 4.3. Limitations

The findings of this review should be interpreted with regard to several limitations. First, our analyses were restricted to published journal articles and we found some indication for publication bias, meaning that analyses on respiratory parameters with insignificant results were reported less often. For instance, some studies described the measurement of single variables without mentioning the according results. As displayed by the integration of reactivity effects, however, a considerable number of studies also reported respiratory measures showing no changes from baseline to task. Second, only 50% of the reviewed studies provided data for a determination of respective effect sizes. Third, the present integration of findings was not weighted according to sample size and quality criteria as required for the statistical data integration in meta-analyses, since larger samples are supposed to increase the precision of findings [37, 103]. This choice was made because the magnitude of effects could be quantified only in 50% of the studies, suggesting a qualitative comparison. But we followed the recommendations by Durlak [89] to adjust the obtained effect sizes for small sample bias. Fourth, comparability of the reviewed studies is limited due to heterogeneous study samples and possible differences regarding the motivation of participants (experimental context, instructions, and incentives) and study design (randomization of trials with modified task difficulty). Fifth, only a small number of studies mentioned to have included potential confounders (covariates) in their analyses such as age and what possibly could lead to an over- or underestimation of effects. Sixth, only a few studies mentioned whether participants were allowed to switch from nasal to oral respiration or vice versa. We assume, however, that studies using methods with a direct sampling from nose and/or mouth instructed individuals accordingly to breathe only through their nose or mouth within the experimental periods. Finally, except for RR, TV, and MV, only few studies were available reporting data for addressing the present research question. As a consequence, it was further not possible to investigate whether respiratory responses vary as a function of different types of cognitive processing.

### 4.4. Conclusive Summary and Implications for a Research Agenda

The primary aim of this study was to investigate whether respiratory parameters are useful indicators of cognitive load in addition to other physiological correlates and well-established performance and self-report measures. We found evidence that RR is a sensitive measure of operator load which can be obtained easily and inexpensively by using a strain gauge. While at first sight TV and MV may contain little additional information, correlated variability in RR, sigh frequency, petCO_2_, VO_2_, and VCO_2_ are promising measures for research and application purposes as they appear to be sensitive to cognitive load and, furthermore, reflect some of the physiological and psychological processes underlying task-related changes in respiratory behavior. However, this review also revealed that the database for evaluating these measures is rather poor in quantity and quality and that most studies are restricted to the traditional measurement of RR and TV.

Since a further motive underlying this study was to contribute to the relatively limited knowledge about breathing under cognitive load, we derived some general recommendations for future research as based on a proper understanding of the respiratory system. This is a complex, multilayered, integrated, and highly versatile system serving to maintain appropriate partial pressures of O_2_ and CO_2_ in the blood to accommodate both metabolic and behavioral demands. At the same time, respiration is intricately involved in speech production. Breathing regulation hence serves stability but also allows flexibility to quickly adapt to internal and external homeostatic challenges. The respiratory system at rest is considered a dynamic steady state, which is characterized by different types of variability and occasionally requires “resetting” which apparently is accomplished by sighing [22]. Importantly, breathing is also largely driven by feedforward regulation, meaning that the system anticipates perturbations (i.e., discrepancies from normative values) and corrects them before they occur [95, 104].

This perspective has a number of implications for the use of respiration to assess cognitive load. First, baseline recordings during which an episode of cognitive load is anticipated may already indicate a certain degree of anticipatory arousal and reduce possible effects of the cognitive load manipulation. This problem could be solved by randomization of baseline and task conditions and by including a “Vanilla baseline” with a low demanding cognitive task, occupying working memory and thus reducing anticipatory arousal [105].

Second, RR alone provides only very partial information about the dynamic changes of the respiratory system. Underlying drive and timing mechanisms such as central inspiratory drive and inspiratory duty cycle may be much more sensitive to cognitive and emotional demands [106]. Moreover, RR alone does not signal whether ventilatory response is adaptive or maladaptive. An increase in rate may be adjusted by a decrease in TV to maintain appropriate breathing. However, absence of appropriate compensation by TV may result in overbreathing which leads to a decrease in etCO_2_. As a consequence, the combined assessment of RR, MV (the product of RR and TV), and gas exchange parameters, particularly etCO_2_, provides a more integrated account of respiratory responses to cognitive load. An additional benefit is that etCO_2_ allows assessing whether the response to cognitive load is in accordance with or in excess of metabolic requirements. The latter state (hypocapnia) is of particular relevance for cognitive load because hypocapnia is associated with reduced cerebral blood flow and possibly impaired cognitive performance [107–109]. In this respect, a more integrated assessment may help to distinguish between cognitive load and stress. In the first case, respiratory changes are supposed to be task-related and support adequate performance, while in the latter case, stress being linked with concerns about threats and protection of the self, respiratory changes may exceed task-related metabolic need [110, 111].

Third, recent evidence suggests that general and specific parameters of respiratory variability allow measuring stability and flexibility of the respiratory system in response to cognitive load [22, 112]. Specifically, variability in RR has been shown to decrease during sustained attention as induced by a task with a single behavioral response set, while during a mental arithmetic, decreased autocorrelations and increased random variability in respiration have been found [20, 86]. Moreover, the need of the respiratory system to reset has been found to differ between sustained attention and mental arithmetic, as manifested by a higher frequency of sighing during or after the task, respectively [20]. These findings suggest that variability of the respiratory system is sensitive to different types of cognitive load and that it is useful to decompose the concept of cognitive load into basic components. A systematic approach to basic cognitive processes might also clarify current inconsistencies in respiratory correlates of cognitive load as revealed, for instance, by this review. To this end, elementary cognitive tasks which require a small number of cognitive processes such as joystick tracking, card-sorting, or response choice tasks could be used in single as well as multiple task configurations, manipulated at different levels of task difficulty to study respiration in response to increasing cognitive load (see also [113]).

Fourth, we suggest taking individual differences into account. Emotional states and personality traits may play an important role in how a demanding cognitive task is appraised, how coping resources are evaluated, and how the individual responds [114–118]. Specific combinations of individual and task-related characteristics may elicit different respiratory patterns, possibly ranging from a strained breathing pattern (characterized by breathing inhibition with elevated etCO_2_) when preparing for highly attentive episodes [119] to hypocapnic hyperventilation when preparing to cope with demanding situations by energy mobilization [120, 121]. A comprehensive assessment, involving the interaction of physiological and person-related measures, is hence required for a better understanding of individual differences in the physiological response to cognitive load.

Finally, an integrative assessment of respiratory measures would be enriched by simultaneously taking cardiac measures into account, because the respiratory system exerts considerable influence on cardiac functioning [122, 123]. Certainly, cardiac measures have a long tradition in research on mental load assessments, which recently predominantly focuses on heart rate variability (HRV). However, beyond studying respiratory sinus arrhythmia (being a major source of HRV, see [124]), concurrent respiratory assessment has largely been neglected. Although the debate on the usefulness of correcting for respiration when assessing HRV has not yet resulted in clear conclusions (see [125–128]), an integrated approach would provide a more elaborate database to detect and interpret psychophysiological responses to cognitive load. Obviously, the parallel assessment of cardiac and respiratory parameters would also impact data-analytic strategies. Analyses could be performed by applying classical multivariate statistics (if assumptions are met) or, for a more detailed examination, by time series data analysis such as change point detection methods (e.g., [129]), transfer function analysis (e.g., [130]), or similar methods that have been employed to study the structure of cardiorespiratory coupling [131–133].

## Figures and Tables

**Table 1:** Characteristics of selected studies (*N* = 54).

	Number of studies	% of studies
Sample		
Average size (range)	32 (7–132)	
Males	21 (3–64)	
Females	20 (1–68)	
Characteristics		
Male only	14	25.93
Female only	3	5.56
Mixed gender	37	68.52
Mean age in yrs (range)	27.08 (18–80)	
Manipulation (type of cognitive task)		
Mental arithmetic	17	25.93
Stroop interference	5	9.26
Memory	6	11.11
Reasoning	6	11.11
Psychomotor	5	9.26
Multitasking	14	25.93
Choice reaction time	2	3.70
Attention	9	16.67
Vigilance	2	3.70
Respiratory measures		
Respiratory rate (RR)	54	100.00
Tidal volume (TV)	24	44.44
Minute ventilation (MV)	9	16.67
Inspiratory time (*T*_*i*_)	1	1.85
Expiratory time (*T*_*e*_)	0	0.00
Inspiratory/expiratory ratio (*T*_*i*_/*T*_*e*_)	1	1.85
Mean inspiratory flow rate (TV/*T*_*i*_)	1	1.85
Inspiratory duty cycle (*T*_*i*_/*T*_tot_)	3	5.56
Expiratory volume (*V*_*e*_)	1	1.85
Contribution of ribcage breathing to *V*_*i*_ (% RCi)	2	3.70
Sigh rate (SR)	2	3.70
Respiratory variability		
Variance of RR (Var (RR))	1	1.85
Coefficient of variation of RR (CV (RR))	3	5.56
Autocorrelation of RR (AR (RR))	3	5.56
Coefficient of variation of TV (CV (TV))	2	3.70
Autocorrelation of TV (AR (TV))	2	3.70
Coefficient of variation of MV (CV (MV))	2	3.70
Autocorrelation of MV (AR (MV))	2	3.70
Partial pressure of end-tidal CO_2_ (petCO_2_)	4	7.41
O_2_ consumption (VO_2_)	4	7.41
CO_2_ production (VCO_2_)	4	7.41
Respiratory exchange ratio (RER)	2	3.70
Apparatus		
Spirometry	7	12.96
Respiratory inductive plethysmography	15	27.78
Strain gauge	19	35.19
Impedance plethysmography	4	7.41
Impedance cardiography	3	5.56
Capnography	6	11.11
Metabolic analyzer	2	3.70

**Table 2:** Overview of selected studies (*N* = 54) for reviewing respiration in response to cognitive task load.

Reference	Year	*N*	Manipulation		Outcome measures	Comments on methodology
			Task type	Period analyzed (s)		
Allen and Crowell [42]	1989	51	ATT, MA	180	RR, TV, MV, VO_2_, VCO_2_	
Althaus et al. [43]	1998	32	MEM	390	RR	
Backs et al. [44]	2003	15	MT	30	RR, TV	Feedback
Backs et al. [45]	2000	27	MT	180	RR	
Backs et al. [46]	1994	12	PM	180	RR, TV	
Backs and Seljos [47]	1994	24	MEM	240	RR, TV, VO_2_, VCO_2_	Verbal response, feedback
Barbosa et al. [38]	2010	17	SI	n/a	RR	Verbal response (one condition)
Beda et al. [39]	2007	25	MA	300	RR (RP), TV, *T*_*i*_/*T*_*e*_	Verbal response (one condition)
Bernardi et al. [40]	2000	12	MA	180	RR, MV	Verbal response (one condition)
Brookings et al. [48]	1996	8	MT	300	RR, TV	
Brouwer et al. [49]	2014	35	MEM	120	RR (RP)	Feedback
Burleson et al. [50]	1998	24^a^	MA	360	RR, TV	Verbal response, feedback
De Visser et al. [51]	1995	43^a^	MEM	600	RR	Feedback
Delistraty et al. [52]	1991	30	MA	60	RR, TV, MV, VO_2_, VCO_2_, RER	
Dijksterhuis et al. [53]	2011	22	MT	n/a	RR	
Duschek et al. [54]	2009	28	ATT	280	RR	
Ettema and Zielhuis [55]	1971	24	ATT	60	RR	
Fairclough et al. [56]	2005	30	MT	240	RR	
Fournier et al. [57]	1999	10	MT	180	RR, TV	
Grassmann et al. [58]	2015	61	MT	300	RR, CV (RR), AR (RR), petCO_2_	
Herbert et al. [59]	2010	38	MA	300	RR	Feedback
Hoshikawa and Yamamoto [60]	1997	8	SI	630	RR, TV, MV	Feedback
Houtveen et al. [61]	2002	22	MT	240	RR, petCO_2_	Feedback
Karavidas et al. [62]	2010	7	MT	300	RR, TV, MV	
Kodesh and Kizony [63]	2014	23	RS	30	RR, TV, *V*_*e*_, VO_2_	
Kuehl et al. [64]	2015	10^a^	ATT	300	RR	
Lackner et al. [65]	2010	20	ATT, MA	300	RR	
Mehler et al. [66]	2009	111	MT	120	RR	
Melis and van Boxtel [67]	2007	52	RS	270–584	RR	
Niizeki and Saitoh [68]	2012	20	MA	180	RR	Feedback
Nilsen et al. [69]	2007	44	CRT	600	RR	Feedback
Novak et al. [70]	2012	24	MA, PM, MT	300	RR, Var (RR)	Verbal response, feedback
Overbeek et al. [71]	2014	83	MEM	150	RR	
Papadelis et al. [72]	2007	10^a^	MT	60	RR	
Pattyn et al. [73]	2010	20	SI	120	RR, TV, *T*_*i*_/*T*_tot_	
		12^b^	SI	120	RR, TV, *T*_*i*_/*T*_tot_	
Pattyn et al. [74]	2008	21	VIG	1800	RR, TV	
Pruneti and Boem [75]	1995	23^a^	RS	n/a	RR	
Roman-Liu et al. [76]	2013	15	ATT, VIG	240	RR	
Roy and Steptoe [77]	1991	10^a^	MA	300	RR	
Schleifer et al. [78]	2008	23	MA, ATT	360	RR, petCO_2_	Feedback (MA condition)
Silvia et al. [79]	2013	36	CRT	300	RR	
Sloan et al. [41]	1991	10	MA	240	RR	Verbal response (one condition)
Sloan et al. [80]	1995	22	MA, SI	240	RR	Feedback
Steptoe et al. [81]	1997	132^a^	RS, PM	300	RR, TV	
Steptoe et al. [82]	1996	132^c^	RS, PM	300	RR, TV	
Troubat et al. [83]	2009	20	RS	300	RR, TV, VO_2_, VCO_2_, RER	
Veltman [84]	2002	20	MT	n/a	RR, TV	
Veltman and Gaillard [85]	1998	12	MT	240	RR, TV, *T*_*i*_	Feedback
Vlemincx et al. [20]	2011	43	ATT, MA	360	RR, TV, MV, %RCi, SR, CV (RR), AR (RR), CV (TV), AR (TV), CV (MV), AR (MV)	Feedback (MA condition)
Vlemincx et al. [86]	2012	47	MA, ATT	240	RR, TV, MV, %RCi, SR, CV (RR), AR (RR), CV (TV), AR (TV), CV (MV), AR (MV)	Feedback (MA condition)
Vögele and Steptoe [87]	1992	37	MA, PM	300	RR	Feedback
Wetzel et al. [88]	2006	80	MA	60	RR, TV	Verbal response, feedback
Wientjes et al. [7]	1998	44	MEM	300	RR, TV, MV, TV/*T*_*i*_, *T*_*i*_/*T*_tot_, petCO_2_	Feedback (one condition)

*Note*. ^a^Control group/condition, ^b^sample of second experiment reported in Pattyn et al. (2010) [73], ^c^same sample as Steptoe et al. (1997) [81]; MA: mental arithmetic; SI: Stroop interference; MEM: memory; RS: reasoning; PM: psychomotor; MT: multiple task; CRT: choice reaction time; ATT: attentional; VIG: vigilance; RR: respiratory rate; RP: respiratory period (inverted direction of significant effects was used to integrate findings with RR); TV: tidal volume; MV: minute ventilation; *T*_*i*_: inspiratory time; *T*_*i*_/*T*_*e*_: inspiratory/expiratory ratio; TV/*T*_*i*_: mean inspiratory flow rate; *T*_*i*_/*T*_tot_: inspiratory duty cycle; *V*_*e*_: expiratory volume; % RCi: contribution of ribcage breathing to inspiratory volume; SR: sigh rate; Var: variance; CV: coefficient of variation; AR: autocorrelation; petCO_2_: partial pressure of end-tidal carbon dioxide; VO_2_: oxygen consumption; VCO_2_: carbon dioxide production; RER: respiratory exchange ratio.

**Table 3:** Overview of respiratory changes in response to reviewed cognitive tasks.

	Changes from baseline to task (*N* = 54)	Changes with increasing task difficulty (*n* = 14)	Reactivity over time/trials (*n* = 6)
RR	↑	↑	↓—
TV	—	—	(—)
MV	↑	(—)	
*T*_*i*_	(↓)	(—)	
*T*_*i*_/*T*_*e*_	(—)		
TV/*T*_*i*_	(↑)		
*T*_*i*_/*T*_tot_	(↑—)		
*V*_*e*_	(↑)		
% RCi	(—)		
SR	(↑—)		
Respiratory variability			
Var (RR)	(↓—)	(—)	
CV (RR)	(—)		(—)
AR (RR)	↓		(—)
CV (TV)	(↑)		(—)
AR (TV)	(—)		(—)
CV (MV)	(—)		(—)
AR (MV)	(—)		(—)
petCO_2_	↓		
VO_2_	↑	(—)	
VCO_2_	(↑)	(↑)	
RER	(↑↓)		

*Note.* ↑: increase; ↓: decrease; —: no change. A combination of two characters indicates that the corresponding effects were reported by an equal number of studies. Parentheses indicate a database of less than three studies for increase, decrease, no change, or mixed effects, respectively. RR: respiratory rate; TV: tidal volume; MV: minute ventilation; *T*_*i*_: inspiratory time; *T*_*i*_/*T*_*e*_: inspiratory/expiratory ratio; TV/*T*_*i*_: mean inspiratory flow rate; *T*_*i*_/*T*_tot_: inspiratory duty cycle; *V*_*e*_: expiratory volume; % RCi: contribution of ribcage breathing to inspiratory volume; SR: sigh rate; Var: variance; CV: coefficient of variation; AR: autocorrelation; petCO_2_: partial pressure of end-tidal carbon dioxide; VO_2_: oxygen consumption; VCO_2_: carbon dioxide production; RER: respiratory exchange ratio.

## Appendix

See Table 2 and Figure 1.
